# Gestational Choriocarcinoma Presenting with Lacrimal Gland Metastasis: A First Reported Case

**DOI:** 10.1155/2015/879538

**Published:** 2015-05-20

**Authors:** Naushad A. B. Ahamed, Khalid Sait, Nisreen Anfnan, Khader Farwan, S. H. M. Nizamuddin, Saleh S. Baeesa

**Affiliations:** ^1^Department of Radiology, Faculty of Medicine, King Abdulaziz University, Jeddah 21589, Saudi Arabia; ^2^Gynecology Oncology Unit, Department of Obstetric and Gynecology, Faculty of Medicine, King Abdulaziz University, Jeddah 21589, Saudi Arabia; ^3^Department of Ophthalmology, Faculty of Medicine, King Abdulaziz University, Jeddah 21589, Saudi Arabia; ^4^Division of Neurosurgery, Faculty of Medicine, King Abdulaziz University, P.O. Box 80215, Jeddah 21589, Saudi Arabia

## Abstract

*Background*. Gestational choriocarcinoma (GC) is a recognized clinicopathological subtype of gestational trophoblastic neoplasia that usually metastasizes hematogenously to highly vascular organs like the lung, liver, and brain. However, orbital metastasis to the choroid and lacrimal gland is a rare occurrence. *Case Presentation*. A 21-year-old female presented with headache and left orbital swelling one year after resection of a complete hydatidiform mole followed by adjuvant methotrexate chemotherapy. A metastatic imaging screening revealed multiple metastases in the lungs, brain, and adrenal gland, in addition to the choroid and lacrimal gland. Based on her modified WHO risk factors scoring she was started on chemotherapy and whole brain radiotherapy, which resulted in a complete response. At two-year follow-up, serum b-HCG level was with normal limits; imaging surveillance was uneventful. *Conclusion*. We present the first case of lacrimal gland metastasis in a young girl from GC relapse.

## 1. Introduction

Gestational Trophoblastic Disease (GTD) is a spectrum of diseases characterized by abnormal proliferation of trophoblasts [1]. There are five recognized clinicopathological subtypes, hydatidiform mole (complete or partial), invasive mole, gestational choriocarcinoma (GC), placental site trophoblastic tumor (PSTT), and epithelioid trophoblastic tumor (ETT) [2].

The term Gestational Trophoblastic Neoplasia (GTN) is reserved for the last four in this subgroup as these have a propensity for progression, invasion, and metastasis any may potentially lead to death if untreated [1]. GTN commonly develops following a hydatidiform mole and less commonly following a live birth or abortion [2].

Gestational choriocarcinoma is a malignant disease with marked trophoblastic hyperplasia resulting in direct invasion into the myometrium and endometrium. About 50% of these occur following a hydatidiform mole, with an incidence of 25% each after abortion or tubal pregnancy [3].

Metastasis develops in about 4% of patients following the surgical evacuation of a molar pregnancy; however, the incidence is higher in nonmolar pregnancies [4]. Due to their highly vascular nature, these tumors show an affinity for hemorrhage and commonly metastasize via the hematogenous route to the lungs (80%), vagina (30%), pelvis (20%), liver (10%), and brain (10%) [4, 5].

Metastasis of GC to the orbit is a very rare presentation in the literature, which was recently reviewed [6]. We present the first case of GC presenting with lacrimal gland metastasis.

## 2. Case Report

A 21-year-old female G1P0 had a termination of her first pregnancy by suction evacuation of a complete hydatidiform mole one year ago. During her preoperative and postsuction follow-up, she had persistently elevated serum level (14,476 IU/L) of beta-human chorionic gonadotropin (b-HCG). She was labeled as GTN-stage I and received methotrexate (MTX) chemotherapy according to WHO score of low risk. Eventual normalization of serum b-HCG was achieved after eight cycles of chemotherapy; she was followed up every 4–6 weeks with pelvic ultrasound scan and serum b-HCG level.

She presented with a 4-week history of headache and progressive left periorbital fullness with blurred vision. Her vitals and general physical and genital examination were within normal; there was no abdominal swelling or vaginal bleeding.

**Figure 1:**
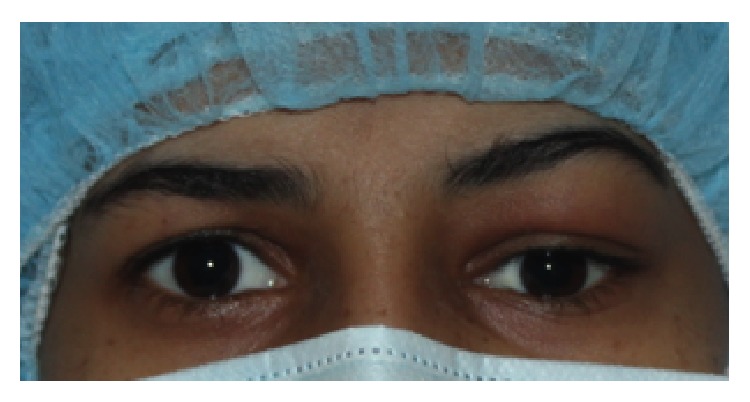
Photographic image of the patient showing the left periorbital swelling.

**Figure 2:**
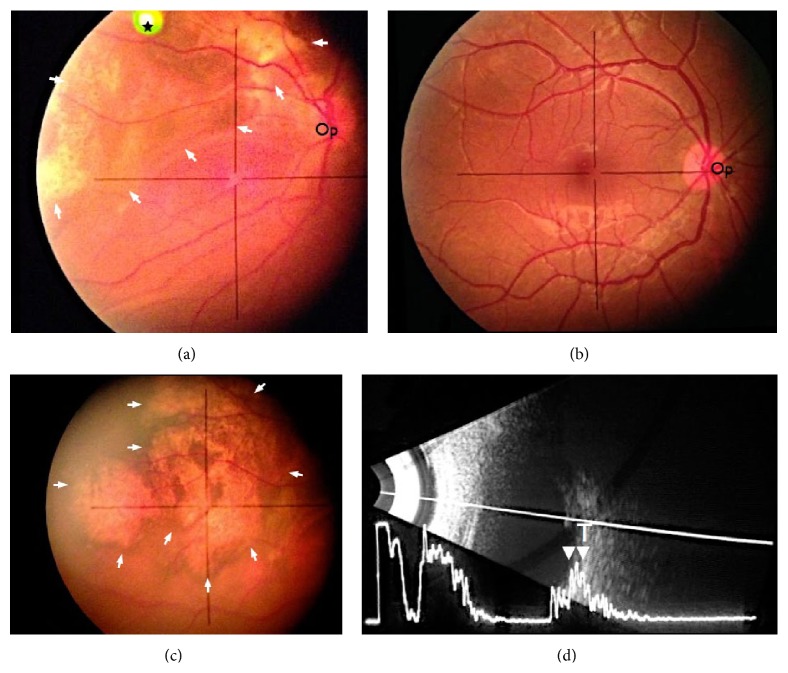
Selected fundoscopy images of the retina (a) at the level of the left optic nerve (Op) and (b) at the level of the right optic nerve (Op); (c) left nasal equatorial retina; (d) ultrasound B-scan with vector A scan. Images (a) and (c) show multiple ill-defined tumors with irregular surface (marked with white arrows in images (a) and (c)). Image (b) shows the comparative normal appearances of the right side. Image (d) shows the retinal lesion (T) with high intralesional reflectivity (white arrowheads). ★ is artifact.

An ophthalmological examination showed the fullness over the left upper lid and mild ptosis (Figure 1). The vision in the affected left eye was 20/200 OS and the vision in the right was normal at 20/20 OD. A fundoscopic examination revealed multiple ill-defined subretinal lesions involving the choroid with the irregular surface. Exudative retinal detachment with shifting subretinal fluid was noted in the inferior fundus. An ultrasound B-scan evaluation (with vector A scan) of the left eye showed multiple choroid lesions with an irregular surface and moderate to high internal reflectivity suggesting choroid mass lesions of vascular nature. There was no evidence for choroidal excavation (Figure 2).

**Figure 3:**
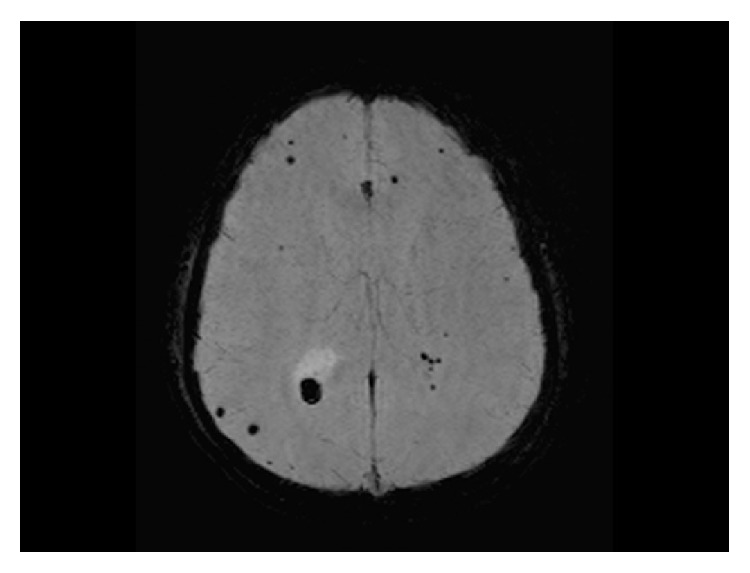
Axial susceptibility-weighted MRI image (TR-50; TE-40) showing multiple “dark” spots of magnetic susceptibility seen scattered in both cerebral hemispheres consistent with hemorrhagic brain metastasis.

Routine laboratory blood investigations were within normal; however, b-HCG was grossly elevated (20,165 IU/L). Metastatic work-up included computed tomographic (CT) studies of the brain, chest, abdomen, and pelvis, which showed multiple hemorrhagic brain lesions, multiple lung nodules, and a small suspected metastatic lesion in the left adrenal gland. There were endometrial infiltrating uterine mass and cystic right adnexal lesion identified as a locoregional recurrence. Magnetic resonance imaging (MRI) scan of the brain revealed multiple small hemorrhagic metastases demonstrated on susceptibility-weighted imaging (SWI) (Figure 3).

**Figure 4:**
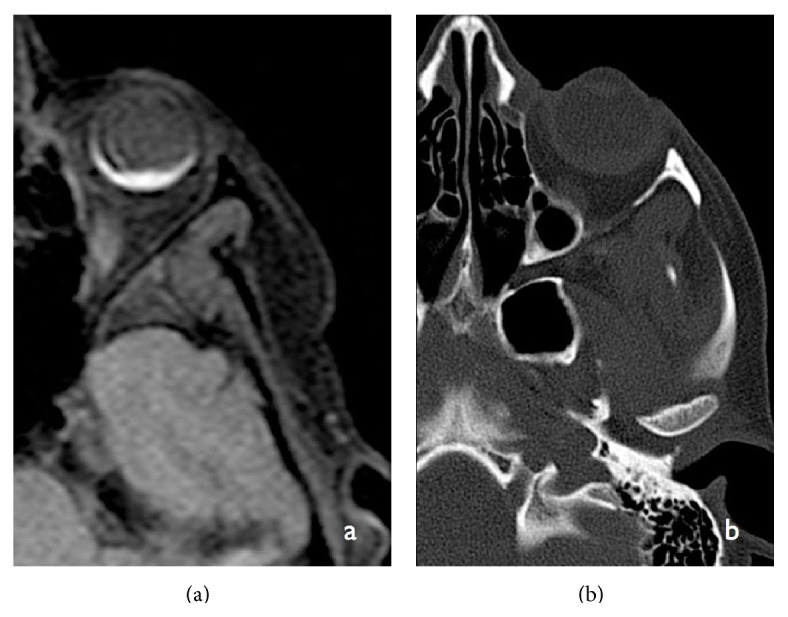
Axial T1-weighted image MRI (a) showing hyperintense lesion in the retinal choroid with a mild exophytic thickening of the uvea. Axial noncontrast enhanced CT section (b) in bone window projection showing the lesion to be of soft tissue densities without calcification.

**Figure 5:**
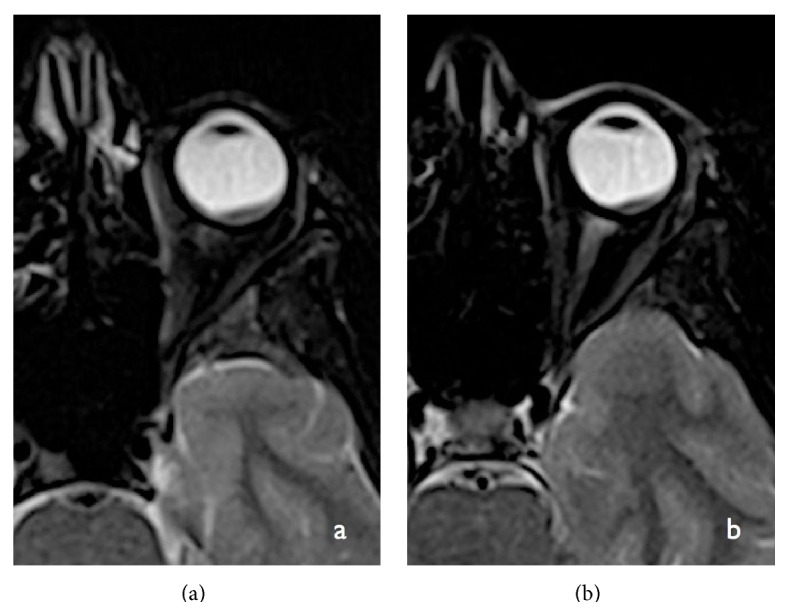
Axial T2-FLAIR MRI image showing that the lesion appears hypointense to gray matter with typical signal intensities of the optic nerve.

**Figure 6:**
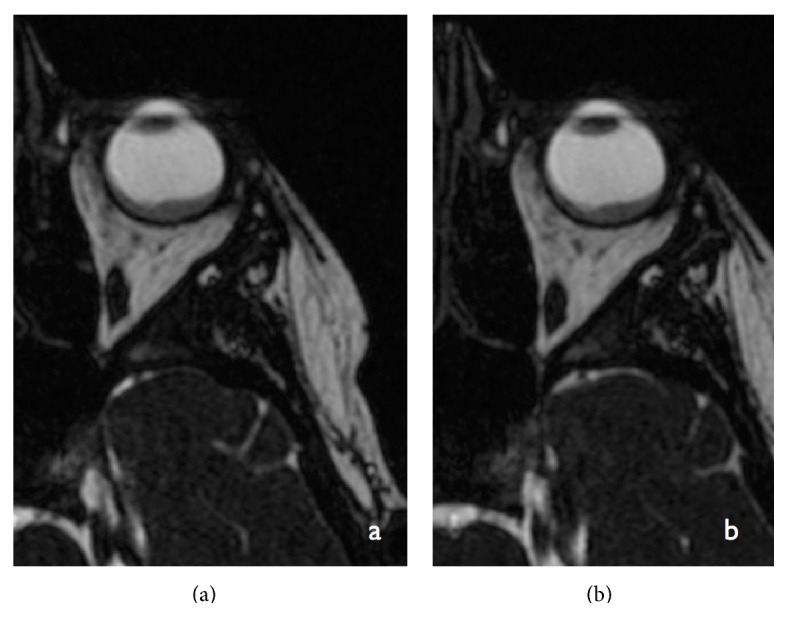
Axial CISS (constructive interference in steady state) MRI images showing that theretinal choroidal lesion shows similar signal characteristics as on T2-weighted images. No calcific signal foci are seen within the lesion. The CISS images provide high spatial resolution and help in radiologically defining the extent of the tumor.

**Figure 7:**
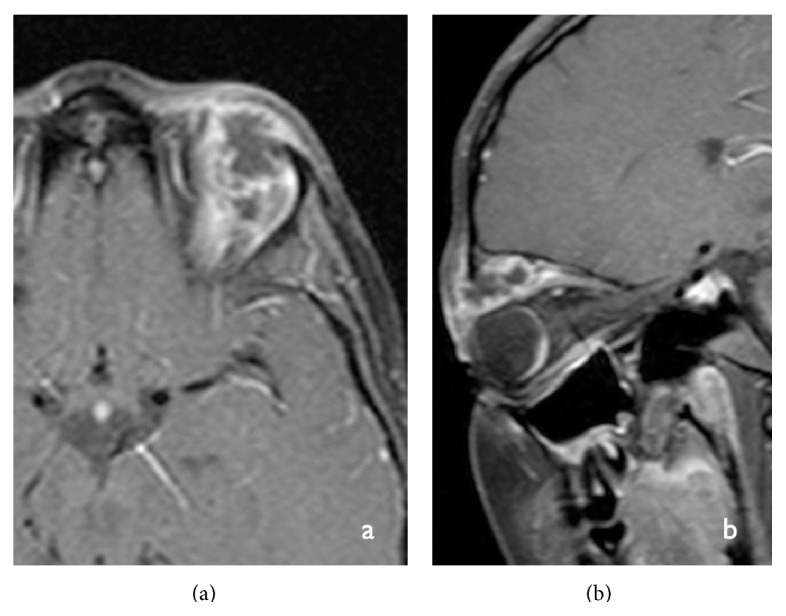
Contrast-enhanced axial (a) and sagittal T1-weighted MRI image showing the choroidal retinal mass and the peripherally enhancing lobulated lesion in the superior lateral extraconal plane centered in the region of the left lacrimal gland.

**Figure 8:**
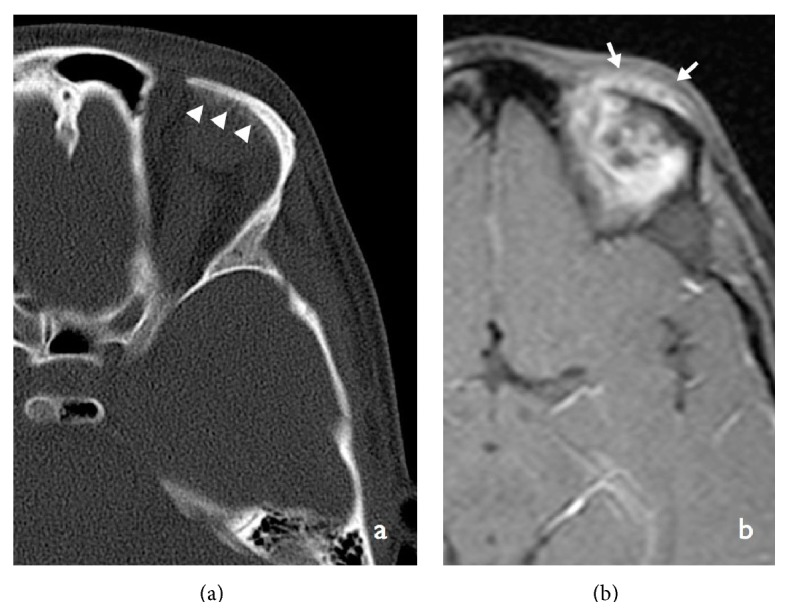
Axial noncontrast enhanced CT section in bone window projection (a) showing periosteal reaction exerted by the lesion on the superior orbital ridge seen as amorphous density. Contrast-enhanced sagittal T1-weighted MRI image (b) showing an amorphous area of enhancement of the lesion.

Orbital MRI scan demonstrated a marginally exophytic lesion in the left eye at the level of choroid adjacent to the optic disc appearing hyperintense to gray matter on T1-weighted images (Figure 4). There was no calcification seen in the slice-matched CT images, thus helping in distinguishing between possible synchronous choroidal pathologies like retinoblastoma. The lesion was hypointense on T2-FLAIR images (Figure 5). The high-resolution axial 3D- constructive interference in steady state (CISS) images showed the full extent of the lesion, which was confined to the retinal choroid with normal appearances of the vitreous chamber of the globe (Figure 6). On imaging, after intravenous gadolinium, the lesion demonstrated homogenous enhancement in keeping with a vascular tumor. Another focal lesion was seen centered in the left lacrimal gland distorting the lacrimal contour (Figure 7). The dark periosteum of the superior orbital ridge was lost in the MRI study, which showed an amorphous enhancement, and a comparison with the CT study revealed faint periosteum thickening (Figure 8). Both findings were in keeping with bone infiltration.

Given the significant disease burden, history of GC, and uterine and systemic imaging findings, the patient was diagnosed as recurrent GC. Based on the modified WHO prognostic scoring system adopted by FIGO for GTN [7], the patient was diagnosed as a high-risk GTN with a score of >7. A multidisciplinary team was involved in management planning for this patient including gynecological oncology, radiology, ophthalmology, and radiation oncology.

The patient was started on EMA-CO chemotherapy regimen (etoposide, methotrexate, folinic acid, actinomycin D, cyclophosphamide, and vincristine). Additionally, the patient was also started on high-dose dexamethasone to reduce cerebral edema exerted by the brain metastasis and received whole brain radiation. She tolerated the treatment well with eventual normalization of her b-HCG, and metastatic imaging scans surveillance was negative for 2-year follow-up.

## 3. Discussion

Ocular metastases, also known as uveal metastasis, occur in about 9 to 10% of all systemic cancer [7–9]. The most common tumors to metastasize to the retina are breast cancer, accounting for 40% of cases, and lungs cancer, particularly small cell carcinoma for about 29% of the cases [8–13]. The choroid is the most common site of such metastatic diseases due to its rich vascularity [14].

Choriocarcinoma has shown metastasis to the orbit, particularly retinal choroid predominantly reported from the male pathological counterparts, testicular choriocarcinoma [13, 15]. A few cases of GC metastasis to the retinal choroid and extraocular muscles have also been reported [6, 14, 16].

Unlike metastatic ocular diseases of other primaries, choriocarcinoma has an affinity for hemorrhage and need an expedited MRI evaluation for diagnosis and management planning. One of the differential diagnoses, which were entertained, was malignant melanoma, which has similar characteristics in T1-MRI scan as a hyperintense lesion. However, they tend to show a characteristic collar button shape and are more exophytic than our study. Radiological appearances of T1-MRI scan of a hyperintense choroidal lesion in the absence of calcification, particularly in the scenarios of other metastasis, helped in the decision-making.

Lacrimal gland metastasis from malignancy is a rarity in itself. Although a site-specific percentage of lacrimal gland metastatic infiltration could not be found, even among the cases with orbital metastasis, mention of lacrimal gland involvement has been few and far between. The common primary tumors metastasizing to the lacrimal glands include esophageal, breast, and renal malignancies [17]. To the best of our knowledge, this is the first case of GC metastasizing to the lacrimal gland.

The International Federation of Gynecology and Obstetrics (FIGO) developed an adapted staging system for GTN, which incorporated the World Health Organization risk factor scoring system for GTN and the previous FIGO anatomical scoring [18]. According to this modified risk-stratification system, our patient was score >7, the highest risk category in the system, based on the number, location, and size of metastases and other prognostic factors, including the level of the serum b-HCG.

Traditionally, the treatment for metastatic GC is first and foremost chemotherapy. Patients with a low-risk disease (score <7) are treated with single-agent therapy, usually methotrexate. Patients classified as having high-risk (score >7) are treated with multiple chemotherapy agents with or without radiation or surgery [19]. Recent literature suggests that patients undergoing a multiagent regimen of EMA-CO have complete response rates of 71–78% and long-term survival rates of 85–94% [19–24].

This is the same applied to orbital GC metastases with EMA/CO chemotherapy regimen without [16] or with local radiotherapy [15]. Our patient has shown complete response to chemotherapy and whole brain radiation with good recovery from the unusual ocular as well as other metastatic lesions.

## 4. Conclusion

Visual impairment and orbital swelling due to orbital metastasis in a patient with GC are rare occurrence and require a high index of suspicion. We emphasize the need of brain and orbital MRI scan in addition to local and systemic radiological evaluation and serum level of b-HCG for assessing the disease burden in all patients with advanced GC for early diagnosis and management planning.

## References

[1] Lurain J. R. (2010). Gestational trophoblastic disease I: epidemiology, pathology, clinical presentation and diagnosis of gestational trophoblastic disease, and management of hydatidiform mole. *American Journal of Obstetrics & Gynecology*.

[2] Sita-Lumsden A., Medani H., Fisher R. (2013). Uterine artery pulsatility index improves prediction of methotrexate resistance in women with gestational trophoblastic neoplasia with FIGO score 5-6. *BJOG*.

[3] Lurain J. R. (1990). Gestational trophoblastic tumors. *Seminars in Surgical Oncology*.

[4] Thomakos N., Rodolakis A., Belitsos P. (2010). Gestational trophoblastic neoplasia with retroperitoneal metastases: a fatal complication. *World Journal of Surgical Oncology*.

[5] Ross S. B., Donald P. G., Berek J. S. (2003). Gestational trophoblastic neoplasia. *Novak's Gynecology*.

[6] Dhrami-Gavazi E., Lo C., Patel P., Galic V., Pareja F., Kazim M. (2014). Gestational choriocarcinoma metastasis to the extraocular muscle: a case report. *Ophthalmic Plastic and Reconstructive Surgery*.

[7] Ascaso F. J., Castillo J. M., García F. J., Cristóbal J. A., Fuertes A., Artal A. (2009). Bilateral choroidal metastases revealing an advanced non-small cell lung cancer. *Annals of Thoracic Surgery*.

[8] LeBedis C. A., Sakai O. (2008). Nontraumatic orbital conditions: diagnosis with CT and MR imaging in the emergent setting. *Radiographics*.

[9] Eliassi-Rad B., Albert D. M., Green W. R. (1996). Frequency of ocular metastases in patients dying of cancer in eye bank populations. *British Journal of Ophthalmology*.

[10] Ferry A. P., Font R. L. (1974). Carcinoma metastatic to the eye and orbit. I. A clinicopathologic study of 227 cases. *Archives of Ophthalmology*.

[11] Arevalo J. F., Fernandez C. F., Garcia R. A. (2005). Optical coherence tomography characteristics of choroidal metastasis. *Ophthalmology*.

[12] Abundo R. E., Orenic C. J., Anderson S. F., Townsend J. C. (1997). Choroidal metastases resulting from carcinoma of the lung. *Journal of the American Optometric Association*.

[13] Purandare N. C., Sanghvi D. A., Thakur M. H. (2008). Choroidal metastasis from non-seminomatous germ cell tumour of the testis. *British Journal of Radiology*.

[14] Pakala S. R., Hollander D. A., O'Brien J. M., Kavanagh M. C. (2006). Choriocarcinoma metastatic to the choroid. *British Journal of Ophthalmology*.

[15] Guber I., Zografos L., Schalenbourg A. (2011). Choroidal metastases in testicular choriocarcinoma, successful treatment with chemo- and radiotherapy: a case report. *BMC Urology*.

[16] Ino K., Mitsui T., Nomura S., Kikkawa F., Mizutani S. (2001). Complete remission of gestational choriocarcinoma with choroidal metastasis treated with systemic chemotherapy alone: case report and review of literature. *Gynecologic Oncology*.

[17] Oworu O., Kyle P., Morton R. (2004). Metastatic oesophageal carcinoma presenting as a lacrimal gland tumour. *British Journal of Ophthalmology*.

[18] Lurain J. R. (2011). Gestational trophoblastic disease II: classification and management of gestational trophoblastic neoplasia. *The American Journal of Obstetrics and Gynecology*.

[19] Escobar P. F., Lurain J. R., Singh D. K., Bozorgi K., Fishman D. A. (2003). Treatment of high-risk gestational trophoblastic neoplasia with etoposide, methotrexate, actinomycin D, cyclophosphamide, and vincristine chemotherapy. *Gynecologic Oncology*.

[20] Bower M., Newlands E. S., Holden L. (1997). EMA/CO for high-risk gestational trophoblastic tumors: results from a cohort of 272 patients. *Journal of Clinical Oncology*.

[21] Kim S. J., Bae S. N., Kim J. H., Kim C. J., Jung J. K. (1998). Risk factors for the prediction of treatment failure in gestational trophoblastic tumors treated with EMA/CO regimen. *Gynecologic Oncology*.

[22] Matsui H., Suzuka K., Iitsuka Y., Seki K., Sekiya S. (2000). Combination chemotherapy with methotrexate, etoposide, and actinomycin D for high-risk gestational trophoblastic tumors. *Gynecologic Oncology*.

[23] Lurain J. R., Singh D. K., Schink J. C. (2006). Primary treatment of metastatic high-risk gestational trophoblastic neoplasia with EMA-CO chemotherapy. *Journal of Reproductive Medicine for the Obstetrician and Gynecologist*.

[24] Aghajanian C. (2011). Treatment of low-risk gestational trophoblastic neoplasia. *Journal of Clinical Oncology*.

